# Transcriptional and functional remodeling of lung-resident T cells and macrophages by Simian varicella virus infection

**DOI:** 10.3389/fimmu.2024.1408212

**Published:** 2024-06-03

**Authors:** Brianna M. Doratt, Delphine C. Malherbe, Ilhem Messaoudi

**Affiliations:** Department of Microbiology, Immunology, and Molecular Genetics, College of Medicine, University of Kentucky, Lexington, KY, United States

**Keywords:** varicella zoster virus, Simian varicella virus, bystander cells, T cells, macrophages, lung, acute viral infection, single-cell transcriptome analysis

## Abstract

**Introduction:**

Varicella zoster virus (VZV) causes varicella and can reactivate as herpes zoster, and both diseases present a significant burden worldwide. However, the mechanisms by which VZV establishes latency in the sensory ganglia and disseminates to these sites remain unclear.

**Methods:**

We combined a single-cell sequencing approach and a well-established rhesus macaque experimental model using Simian varicella virus (SVV), which recapitulates the VZV infection in humans, to define the acute immune response to SVV in the lung as well as compare the transcriptome of infected and bystander lung-resident T cells and macrophages.

**Results and discussion:**

Our analysis showed a decrease in the frequency of alveolar macrophages concomitant with an increase in that of infiltrating macrophages expressing antiviral genes as well as proliferating T cells, effector CD8 T cells, and T cells expressing granzyme A (GZMA) shortly after infection. Moreover, infected T cells harbored higher numbers of viral transcripts compared to infected macrophages. Furthermore, genes associated with cellular metabolism (glycolysis and oxidative phosphorylation) showed differential expression in infected cells, suggesting adaptations to support viral replication. Overall, these data suggest that SVV infection remodels the transcriptome of bystander and infected lung-resident T cells and macrophages.

## Introduction

Varicella is a highly contagious human disease caused by the varicella zoster virus (VZV), a herpesvirus classified into *Human alphaherpesvirus 3* species and a member of the *Orthoherpesviridae* family (1). Varicella causes approximately 140 million cases annually, of which 4.2 million cases are severe and require hospitalization (2). VZV is transmitted from person to person by inhalation of infected saliva droplets or direct contact with fluid from skin lesions. Varicella is usually a mild, self-limiting illness characterized by fever, malaise, and a generalized vesicular rash. Additionally, VZV establishes latent infection in neurons of the sensory ganglia (3–6). Reactivation of VZV due to a decline in immunity with stress, aging, or immunosuppression results in herpes zoster (HZ) (7). HZ can be a debilitating disease, with 10% of affected adults developing postherpetic neuralgia and persistent pain at the site of exanthem lasting greater than 90 days after initial VZV reactivation (8). Currently, 1 million HZ cases are estimated annually in the United States, reaching 1% prevalence among individuals aged 60 years or older (8).

Simian varicella virus (SVV) is a herpesvirus that also belongs to the *Orthoherpesviridae* family, which exclusively infects Old World Monkeys (9). SVV infection in non-human primates shares significant clinical, pathological, immunological, and virological features with VZV infection in humans. For instance, non-human primates vaccinated with VZV are protected against SVV infection mediated in part by a robust neutralizing antibody response (10, 11). Among the various Old World macaque species, rhesus macaques experience a self-limited acute disease that most closely recapitulates the hallmarks of varicella in humans (3, 4, 12–14). Furthermore, SVV can establish latency in rhesus macaques and reactivate following immune suppression or stress, which is like HZ in humans (5, 12, 15, 16).

Following intrabronchial infection of rhesus macaques, SVV infects epithelial, myeloid, and lymphoid cells, rapidly spreading through the lung, resulting in focal tissue damage 7 days post-infection (DPI) (17, 18). The innate antiviral response to SVV in the alveolar space [sampled by bronchoalveolar lavage (BAL)] at 7 DPI is characterized by increased production of proinflammatory mediators, chemokines, and growth factors concomitant with the infiltration of plasmacytoid dendritic cells, monocytes, macrophages, and neutrophils (14). Innate immune responses to SVV are subsequently followed by the activation of the adaptive immune system in the lung with infiltration of memory and proliferating SVV-specific T cells at 7–21 DPI, as well as detection of SVV-specific antibodies in BAL (17–19). Both viral transcripts and memory CD4 and CD8 T cells are detected in the sensory ganglia of SVV-infected macaques as early as 3 DPI (20), and latency is established at 7 DPI in the ganglia (12). Infiltrating CD4 T cells are important to the establishment of latency in the ganglia, as CD4 T-cell depletion results in persistent expression of lytic viral genes in the ganglia (21).

To date, data from clinical and animal model studies suggest that VZV and SVV initially infect respiratory mucosal epithelial and immune cells. Then, infected T cells migrate from the respiratory system to the skin, causing the characteristic varicella exanthem, and to the central nervous system, where the virus establishes a latent infection (7). However, the characteristics and molecular changes occurring within infected cells to facilitate viral dissemination from the respiratory tract to the sensory ganglia remain ill defined. In this study, we investigated transcriptional changes within lymphoid and myeloid cells from BAL collected during acute SVV infection (0–14 DPI) of rhesus macaques at the single-cell resolution.

## Methods

### Animal and sample collection

Archived BAL samples that were collected during prior SVV infection studies were used (**Supplementary Table 1**). Details regarding inoculation, sample collection, and storage as well as ethical approval can be found in the original studies (5, 20, 22). BAL samples were centrifuged, supernatant was isolated, and cells were counted and cryopreserved for later use.

### 10x Genomics 3′ single-cell capture (10x 3′ scRNAseq) and Illumina library preparation

Freshly thawed BAL leukocytes from rhesus macaque infected with SVV (n = 6) were resuspended in phosphate-buffered saline (PBS) with 0.04% bovine serum albumin (BSA) and counted in triplicates on a TC20 Automated Cell Counter (Bio-Rad Laboratories, Hercules, CA, USA). Equal cell numbers of each sample were pooled by timepoint and resuspended to a final concentration of 1,500 cells/μL. Single-cell suspensions were then immediately loaded on the Chromium Controller (10x Genomics, Pleasanton, CA, USA) with a target of 20,000 cells/timepoint. V3 chemistry was used for library generation before being sequenced on an Illumina NovaSeq 6000 with a sequencing target of 30,000 paired gene expression reads per cell.

### SMART-Seq2 libraries

Live macrophages and T cells were sorted from cryopreserved BAL samples obtained from SVV-infected rhesus macaques at 0 and 7 DPI by fluorescence-activated cell sorting (FACS) (n = 3–4/timepoint) into 96-well plates at a rate of 1 cell/well. Then, the cells were subjected to the Smart-Seq2 protocol for RNA extraction and cDNA synthesis, as previously described (23). Before library preparation, qPCR was performed on the cDNA using primers and probes specific for SVV ORF15 in triplicates to identify SVV-infected and bystander cells. Sequences of primers were GTACCCGGTGCAACAGACA (forward) and CCCCAAAGTTGTTCCAATAATCATTCC (reverse), and the probe sequence was 56-FAM/AAA CAC ATA/ZEN/TAC AAG TAT CGG AAG A/1ABkFQ (IDT, Coralville, IA, USA). Cycling conditions were as follows: polymerase activation (95°C, 3 min), 40 cycles of denaturation (95°C, 15 sec), and annealing/extension (60°C, 1 min), held at 4°C. Concordant results across all three wells were required to designate a cell as SVV-infected or SVV-negative. These results were confirmed by running the PCR product on an agarose gel.

### 10x 3′ scRNAseq and SMART-Seq2 analysis

10x 3′ scRNAseq and SMART-Seq2 samples were analyzed as pools for each indicated timepoint. CellRanger Single-Cell Software Suite version 3.0.1 (10x Genomics) or Bowtie2 (24) in the case of the SMART-Seq2 data was used to align and quantify raw reads using the rhesus macaque (*Macaca mulatta*, Mmul_10) reference genome [National Center for Biotechnology Information (NCBI), RefSeqe GCF_003339765] that was concatenated with the SVV [*Cercopithecine alphaherpesvirus 9* (*CeHV-9*)] reference genome (EMBL-EBI AF275348.3). For SMART-Seq2 data, aligned reads were counted using GenomicRanges in a strand-specific manner. Count matrices were generated such that each column is a cell and each row is a gene. Timepoint (0 or 7 DPI) and SVV ORF15 qPCR status were retained within the cell column name for downstream group identification.

Seurat version 3.1.1 was used for downstream processing of aligned reads. Environmental RNA, potential doublets, and dying cells were excluded during initial QC by removal of droplets containing fewer than 400 detected genes, more than 4,000 detected genes, or more than 20% of total mitochondrial gene expression. Data were normalized using the *SCTransform* function (25) and regressed for differential effects of mitochondrial and ribosomal gene expression levels and cell cycle states. The *RunPCA* function was used to perform dimension reduction and obtain the first 30 principal components followed by clustering and visualization using the *FindClusters* and *runUMAP* functions in Seurat. The *FindAllMarkers* function with a log2 fold-change cutoff of 0.5 was used to identify cluster marker genes and assign cell types to individual clusters for the 3′ GEX dataset. Marker genes for all clusters are provided in **Supplementary Table 2**. The frequency of cell clusters is provided in **Supplementary Table 2**. The *FindMarkers* function was used to identify differentially expressed genes (DEGs) between infected and bystander cells at 7 DPI relative to 0 DPI using MAST for the SMART-Seq2 data (26). For gene scoring analysis, gene signatures and pathways in subpopulations were compared using Seurat’s *AddModuleScore* function. Gene lists for each module as well as the fold change of modules relative to DPI 0 are provided in **Supplementary Table 3**. Longitudinal DEGs were identified using the PALMO package and *sclongitudinalDEG* function, which utilizes normalized gene expression modeled as a linear function of time to evaluate the slope and corresponding p-value (27). Genes with a false discovery rate (FDR) value of <0.05 and expressed in >10% of cells were considered significant for this study. Functional enrichment was performed using Metascape (28). All graphs were generated in R.

### MitoTracker and 2-NBDG staining

A total of 5 × 10^5^ freshly thawed total BAL cells (n = 4 animals for the infected group and n = 3 for the control group) were used. For the MitoTracker assay, cells were incubated for 30 min at 37°C in 50 nM MitoTracker red (Invitrogen, Carlsbad, CA, USA) before one wash in dPBS. For the 2-NBDG assay, cells were treated with 100 ng lipopolysaccharide (LPS) and incubated for 1 hour at 37°C before the addition of 60 mM 2-NBDG (Thermo Fisher, Waltham, MA, USA) for 30 min at 37°C. Following either of these steps, cells were stained with CD206 (clone 19.2) and CD3 (clone SP34) monoclonal antibodies and acquired with an Attune NxT Flow Cytometer (Thermo Fisher, USA) and further analyzed using FlowJo 10.5 software (Ashland, OR, USA). Staining positive and negative controls were included for surface markers and MitoTracker or 2-NBDG reagents.

### Metabolic profiling using Seahorse

The cell energy phenotype kit (Agilent Technologies, Santa Clara, CA, USA) was used per the manufacturer’s directions using the XFp extracellular flux analyzer (Agilent Technologies, USA). Briefly, monocytes or T cells were purified using MACS beads (Miltenyi Biotec, Auburn, CA, USA), and 2 × 10^5^ cells (n = 3 pooled) in XF Base Medium supplemented with 1 mM of sodium pyruvate, 10 mM of glucose, and 2 mM of l-glutamine were plated in a cell cartridge and incubated for 1 hour in a 37°C incubator without CO_2_. Baseline oxygen consumption rate (OCR) and extracellular acidification rate (ECAR) were measured followed by an injection of the stressor mix consisting of oligomycin (ATP synthase inhibitor) and carbonil cyanide *p*-trifluoromethoxyphenylhydrazone (FCCP; a mitochondrial uncoupling agent).

The glycolysis rate assay was performed on the Seahorse XFp Flux Analyzer (Agilent Technologies, USA) following the manufacturer’s instructions. Briefly, 2 × 10^5^ purified monocytes or T cells (n = 3/group pooled) were seeded on Cell-Tak (Corning, New York, NY, USA) coated 8-well culture plates in phenol-free Roswell Park Memorial Institute (RPMI) media containing 2 mM l-glutamine, 1 mM sodium pyruvate, and 5 mM HEPES buffer solution. Cells were cultured in the presence/absence of phorbol myristate acetate (PMA)/ionomycin for 1 hour in a 37°C incubator without CO_2_. Plates were run on the XFp for basal measurements followed by acute injection of Rot/AA (50 μM) and 2-DG (500 mM). All data were analyzed using the Seahorse Wave software (Agilent Technologies, USA).

### Statistical analysis

All statistical analyses were conducted in Prism 10 (GraphPad, La Jolla, CA, USA). Outliers were identified using ROUT analysis (Q = 0.1%) after normality testing using the Shapiro–Wilk test (alpha = 0.05).

For three- and four-group comparisons, the Gaussian assumption was not satisfied, and differences were tested using the Kruskal–Wallis test (alpha = 0.05) followed by Dunn’s multiple hypothesis correction tests. Two-group comparisons were normally distributed and were tested using an unpaired t-test with Welch’s correction. * p < 0.05, ** p < 0.01, *** p < 0.001, **** p < 0.0001, and ns = not significant, unless otherwise indicated.

### Data availability

Data underlying the transcriptome analysis are available under project number PRJNA1091695 within the Sequence Read Archive at the NCBI.

## Results

### Immune response dynamics during acute SVV infection of rhesus macaques

**Figure 1:**
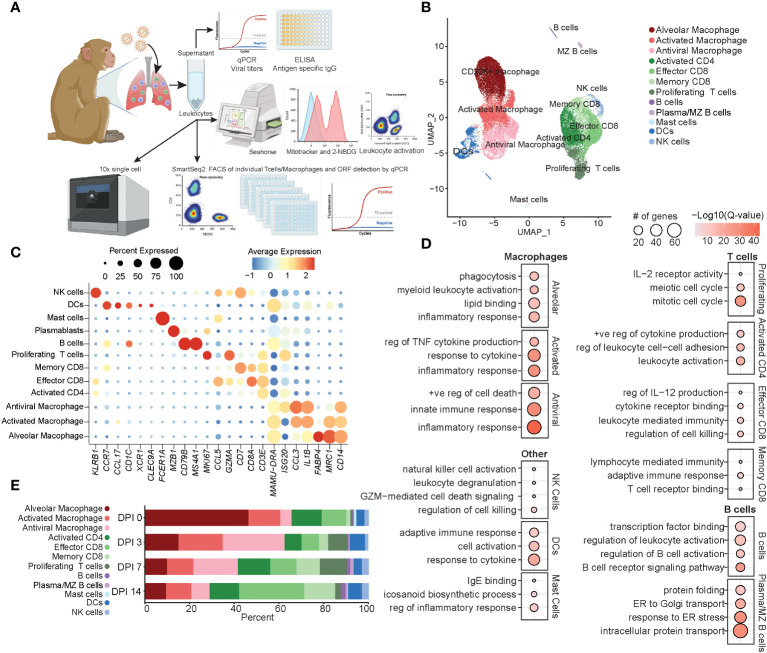
Immune response by total BAL cells during acute SVV infection. **(A)** Experimental design. **(B)** UMAP of 37,095 BAL cells from six SVV-infected rhesus macaques at 0, 3, 7, and 14 DPI. **(C)** Bubble plot of genes used to identify cell populations in panel **(B)** The size of the bubble denotes the percent of cells expressing the marker, and the color denotes the average expression level of the marker. **(D)** Bubble plots of GO terms for cluster marker genes for the indicated cluster. The size of the bubble denotes the number of genes mapping to each GO term, and the color denotes the −log10(Q-value). **(E)** Stacked bar plot of the percent of each cluster at each DPI. BAL, bronchoalveolar lavage; SVV, Simian varicella virus; UMAP, uniform manifold approximation and projection; DPI, days post-infection; GO, gene ontology.

VZV and SVV are respiratory pathogens; however, our understanding of the immune response in the lung during acute infection remains limited. Therefore, we defined longitudinal transcriptional changes in total BAL leukocytes obtained at 0, 3, 7, and 14 DPI with SVV using 10x Genomics 3′ single-cell RNA sequencing (v3) (**Figure 1A**) and cryopreserved BAL samples from our previous studies (**Supplementary Table 1**) (5, 20, 22). The immune response of the rhesus macaques used in these prior studies is summarized in **Supplementary Figure 1**. Briefly, following intrabronchial infection with SVV, viral DNA was detectable at 3 DPI and peaked at 7 DPI before falling below detection at 14 DPI (**Supplementary Figure 1A**). SVV-specific IgG end-point titers (EPT) were observed at 14 DPI, peaked at 21 DPI, and remained stable at up to 96 DPI (**Supplementary Figure 1B**). T-cell proliferation indicated by increased expression of Ki67 was evident as early as 3 DPI and peaked at 14 DPI on average before returning to baseline at 35 DPI (**Supplementary Figure 1C**). The frequency of antigen-specific CD4+ and CD8+ T cells in the BAL-producing TNF-α and IFN-γ peaked at 14 and 28 DPI before slowly declining (**Supplementary Figure 1D**).

Dimensional reduction using uniform manifold approximation and projection (UMAP) identified 12 unique clusters composed of cells from each timepoint (**Figure 1B**; **Supplementary Figure 1E**) based on their expression of specific genes and canonical markers (29–31). We identified three macrophage clusters all expressing *CD14* and *MAMU*-*DRA* that could be further subsetted into alveolar macrophages [*MCR1* (CD206) and *FABP4*], activated macrophages expressing high levels of pro-inflammatory mediators (*IL1B* and *CCL3*), and antiviral macrophages expressing high levels of antiviral genes (*ISG15*, *ISG20*, *IFI16*, and *IRF7*) (**Figures 1B, C**). T-cell subsets included activated CD4 (*ISG20* and *CD3E* without *CD8*), effector CD8 (*CD3E*, *CD8*, and *CCL5*), memory CD8 (*CD3E*, *CD8*, and *CD7*), and finally, a cluster of proliferating T cells expressing high levels of *MKi67* in addition to canonical *CD3E* (**Figures 1B, C**). Additionally, we identified clusters of dendritic cells (DCs) (*CCR7*, *CCL17*, and *MAMU-DRA*), NK cells (*KLRB1*), B cells (*CD79B* and *MS4A1*), marginal zone (MZ)-like/plasmablasts (*MZB1*), and mast cells (*FCER1A*) (**Figures 1B, C**).

Functional enrichment analysis of cluster marker genes revealed that all macrophage marker genes mapped to the gene ontology (GO) term “inflammatory response”, with alveolar macrophage marker genes additionally mapping to “phagocytosis” and “lipid binding” terms, those of antiviral macrophages mapped to “innate immune response”, and those of activated macrophages mapped to “regulation of TNF cytokine production” and “response to cytokine” (**Figure 1D**). As expected, marker genes for the proliferating T-cell cluster mapped to processes involved in cell division (“mitotic cell cycle” and “meiotic cell cycle”) and proliferation/differentiation (“IL-2 receptor activity”) (**Figure 1D**). Genes that defined CD8 T-cell subsets mapped to “regulation of cell killing” as well as “T cell receptor binding” in line with their cytolytic functions, while marker genes of CD4 T cells mapped to “leukocyte activation” and “positive regulation of cytokine production” in line with their helper function (**Figure 1D**). B-cell subset markers mapped to “regulation of B cell activation” and “B cell receptor signaling pathway” (**Figure 1D**). Markers of NK cells mapped to “NK cell activation” and “regulation of cell killing”, while those of DC mapped to “adaptive immune response” and “response to cytokine” (**Figure 1D**). Mast cells were characterized by the expression of genes important for “IgE binding” and “icosanoid biosynthetic process” (**Figure 1D**). The frequency of these cell clusters evolved over the course of acute infection, with a loss of alveolar macrophages at 3 DPI correlating with an initial expansion of antiviral macrophages and DCs at 3 DPI, followed by an expansion of the proliferating T cells at 7 DPI. Finally, at 14 DPI, an expansion of effector and memory CD8 T cells was observed compared to previous timepoints (**Figure 1E**). In summary, these data showed SVV acute infection results in dramatic remodeling of the alveolar space with decreased frequency of alveolar macrophages, while that of infiltrating macrophages and T cells increased.

### Longitudinal response by myeloid cells during acute SVV infection in rhesus macaques

**Figure 2:**
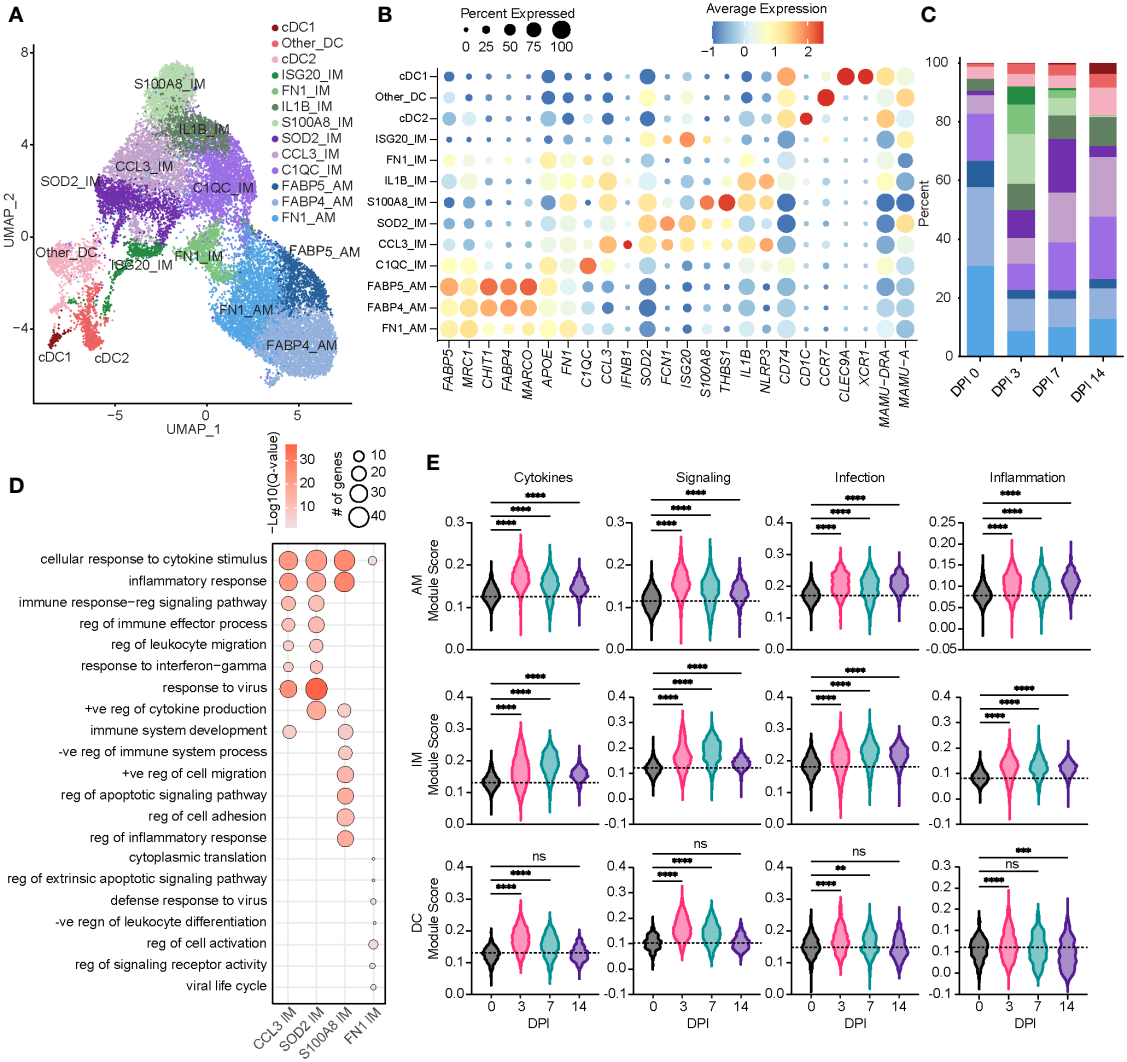
Acute SVV infection recruits infiltrating macrophages to the lung concomitant with a loss of alveolar macrophages. **(A)** UMAP of 21,748 myeloid cells. **(B)** Bubble plot of genes used to identify cell populations in panel **(A)** The size of the bubble denotes the percent of cells expressing the marker, and the color denotes the average expression level of the marker. **(C)** Stacked bar plot of the percent of each cell cluster at each DPI. **(D)** Bubble plots of GO terms for cluster marker genes in the indicated IM clusters. The size of the bubble denotes the number of genes mapping to each GO term, and the intensity of color denotes the −log10(Q-value). **(E)** Violin plots of AM, IM, and DC module scores across time. Dotted line indicates the average value at DPI 0. ns, not significant; **p < 0.01, ***p < 0.001, ****p < 0.0001. SVV, Simian varicella virus; UMAP, uniform manifold approximation and projection; DPI, days post-infection; GO, gene ontology; IM, infiltrating macrophage; AM, alveolar macrophage; DC, dendritic cell.

**Figure 3:**
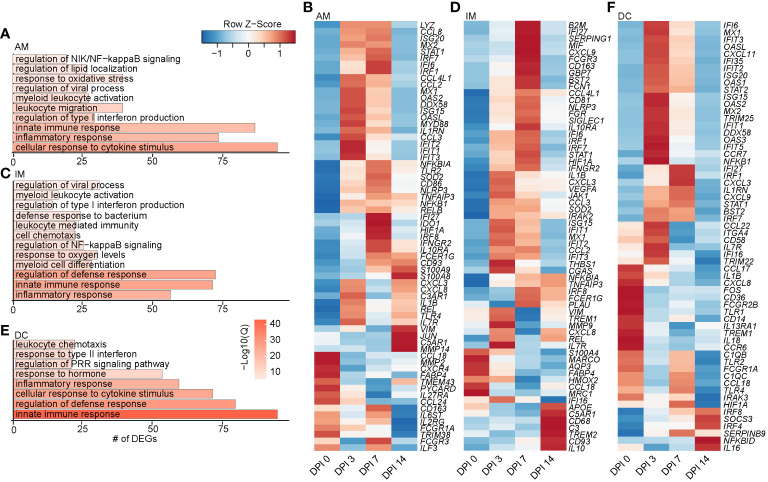
Longitudinal transcriptomic changes within myeloid BAL cells. Bar plots of GO terms associated with DEGs in the AM **(A)**, IM **(C)**, and DC **(E)** clusters over time. Length of the bar indicates the number of genes mapping to each GO term, and the intensity of color denotes the −log10(Q-value). Heatmaps of the average expression of indicated DEGs associated with GO terms in AM **(B)**, IM **(D)**, and DC **(F)** clusters over time. BAL, bronchoalveolar lavage; GO, gene ontology; DEGs, differentially expressed genes; AM, alveolar macrophage; IM, infiltrating macrophage; DC, dendritic cell.

Next, we subsetted and re-clustered myeloid cells to perform a detailed analysis of their transcriptional response to SVV infection (**Figures 2**, **3**). We identified 13 unique clusters (**Figure 2A**): three alveolar macrophage (AM) clusters expressing varying levels of *FABP5*, *FABP4*, *CHIT1*, *MARCO*, or *FN1*; seven infiltrating macrophage (IM) populations all expressing *CD14* and *MAMU-DRA*, but varying levels of *ISG20*, *FN1*, *IL1B*, *S100A8*, *SOD2*, *CCL3*, and *C1QC*; and three DC clusters expressing varying levels of *CD74*, *CLEC9A*, *XCR1*, and *CD1c* [conventional (c)DC1 and cDC2 and other DCs] (**Figure 2B**). At 3 DPI, a decrease in the frequency of all AM populations was accompanied by an increase in the frequency of IM subsets (**Figure 2C**). The frequency of other DCs increased at 3 DPI, while an expansion of the cDC1 (expressing high levels of *XCR1*) population was observed at 14 DPI (**Figure 2C**).

Based on a significant increase in the relative abundance of the IM subpopulations, we further investigated these populations. The *S100A8* IM subset expanded dramatically at 3 DPI before disappearing at 14 DPI (**Figure 2C**). The marker genes of this subset mapped to the GO terms associated with inflammatory and cytokine responses (*S100A8*, *S100A9*, *IL1B*, *STAT3*, *NFKB1*, and *IFNGR2*) (**Figure 2D**). The *SOD2* IM cluster peaked at 7 DPI, while the *CCL3* IM cluster frequency increased at 7 DPI and remained high at 14 DPI (**Figure 2C**). In addition to inflammatory processes and migration, marker genes for the *CCL3* and *SOD2* IM clusters strongly enriched to “response to virus” (**Figure 2D**; **Supplementary Figures 1F–H**). The scores of gene modules associated with cytokine signaling and production, cellular signaling, and infection increased within macrophage clusters at 3 DPI and remained increased at 14 DPI compared to 0 DPI (**Figure 2E**). However, scores of these gene modules were increased at 3 and 7 DPI within DC populations before returning to baseline at 14 DPI (**Figure 2E**).

Next, we identified genes within each cell type that experienced significantly altered expression over time using the PALMO package. DEGs within AM clusters mapped to “myeloid leukocyte activation” (*JUN*, *STAT1*, *CXCL3*, *FCGR3A*, and *MYD88*), viral, cytokine response processes (*TLR2*, *TLR4*, and *IFNGR2*), and regulation of NF-κB signaling (*NFKBIA*) (**Figures 3A,B**). Expression of most of these DEGs peaked at 3 or 7 DPI before returning to baseline. However, expression of alarmins (*S100A8* and *S100A9*), transcription factors (*JUN*), and genes involved in complement system (*C5AR1*), chemotaxis (*VIM*), and tissue remolding (*MMP14*) peaked at 14 DPI. Downregulated DEGs are involved in inflammation (*PYCARD*), immune signaling (*TMEM43*), and receptors important for endocytosis and antibody-dependent cellular cytotoxicity (ADCC) of virus-infected cells (*CD163*, *FCGR1*, and *FCGR3*) (**Figure 3B**).

As described for the AM subset, DEGs within the IM clusters peaked at 3–7 DPI, with a few peaking at 14 DPI, such as *IL10* (anti-inflammatory cytokine), *CD93* (phagocytosis), *CD68* (recruitment and activation), and *APOE* (lipid transport). Downregulated DEGs included *MARCO* (innate antimicrobial system), *MRC1* (antiviral response), *CCL18* (T-cell chemotaxis and lung homeostasis), and *HMOX2* (response to oxygen levels) (**Figures 3C, D**). DEGs within the DC clusters also mapped to defense and innate immune responses (*C1QC*, *ISG20*, *IFIT5*, *IL1B*, *IL18*, *CCL22*, *TLR4*, *IRF7*, and *IRAK3*) (**Figures 3E, F**). Like macrophage subsets, expression of the majority of DEGs in DC subsets peaked at 3–7 DPI. A few DEGs peaked 14 DPI, including genes involved in interferon and inflammatory response (*IRF8*, *IRF4*, and *NFBKID*), immune regulation (*SOCS3*), and inhibitory molecules (*IL16* and *SERPINB9*) (**Figure 3F**). Overall, these data show that a robust antiviral transcriptional response to SVV occurred in the lung of acutely infected macaques by both alveolar and infiltrating macrophages.

### Longitudinal response by lymphocytes during acute SVV infection in rhesus macaques

**Figure 4:**
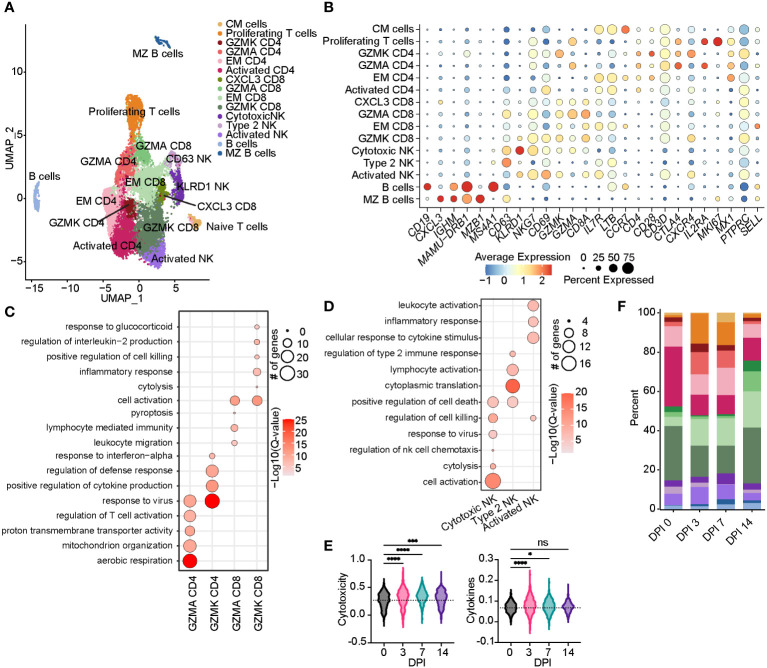
Acute SVV infection promotes lymphoid proliferation and expansion of CD8 T cells within the BAL. **(A)** UMAP of 15,347 lymphocytes. **(B)** Bubble plot of genes used to identify cell clusters in panel **(A)** The size of the bubble denotes the percent of cells expressing the marker, and the color denotes the average expression level of the marker. **(C,D)** Bubble plots of GO terms for cluster marker genes in CD4/8 T cell **(C)** and NK cell clusters **(D)**. The size of the bubble denotes the number of genes mapping to each GO term, and the intensity of color denotes the −log10(Q-value). **(E)** Violin plots of NK cell module scores across time. Dotted line indicates the average value at DPI 0. **(F)** Stacked bar plot of the percent of each cell cluster from the total cells at each timepoint post-infection. ns, not siginificant, *p < 0.05, ***p < 0.001, ****p < 0.0001. SVV, Simian varicella virus; BAL, bronchoalveolar lavage; UMAP, uniform manifold approximation and projection; GO, gene ontology; DPI, days post-infection.

We re-clustered lymphoid cell populations to decipher their detailed transcriptional response to acute SVV infection in rhesus macaques. This analysis resulted in the identification of 15 lymphoid populations (**Figure 4A**). Two clusters of B cells emerged: B cells (*CD19*, *MAMU-DRB1*, and *MS4A1*) and MZ-like/plasmablasts (*CXCL3*, *IGHM*, and *MZB1*) (**Figures 4A, B**). We identified 10 clusters of T cells, including a central memory T cells (*IL7R*, *LTB*, and *CCR7*), effector memory (EM) CD8 (*CD8A*) and CD4 (*IL7R* and *LTB*), *CXCL3* CD8, *GZMK* CD8 and CD4 (*CXCR4*), *GZMA* CD8 and CD4, activated CD4 (*CD4*, *LTB*, and *CD69*), and a subset of proliferating T cells (*IL2RA* and *MKI67*) (**Figures 4A, B**). Marker genes for GZMA and GZMK CD4 clusters mapped to “response to virus” (*OASL*, *APOBEC3F*, *MX1*, and *MX2*). Moreover, genes that defined the GZMA CD4 T-cell cluster mapped to “aerobic respiration” and “mitochondrial organization” (*COX1*, *IDH2*, and *NDUFC2*), while those of the GZMK CD4 T-cell cluster mapped to “regulation of cytokine production” (*LAG3*) and “defense response” (*CXCR4*, *STAT1*, *IFI6*, and *HERC5*) (**Figure 4C**; **Supplementary Figure 2A**). The GZMK CD8 T-cell cluster mapped to “inflammatory response” (*TNFAIP3* and *CCL5*), “cell killing” (*PRF1*), and “interleukin-2 production”, while the GZMA CD8 T-cell cluster mapped to “leukocyte migration” and “pyroptosis” (*PYCARD*) (**Figure 4C** and **Supplementary Figure 2B**). We detected three clusters of NK cells, including an activated NK cell cluster (*NKG7* and *CD69*), a second cluster of putative type 2 NK cells (32) expressing high levels of *CD63*, and a third cluster of highly cytotoxic NK cells expressing high levels of *KLRD1* and *NKG7* (**Figures 4A, B**). Marker genes within the three NK cell clusters mapped to “response to virus”, “regulation of cell killing”, and “lymphocyte activation” (*CD81*, *LAT2*, *CD7*, and *CD44*) (**Figure 4D**; **Supplementary Figure 2C**). In addition, we observed an increase in the scores of gene modules associated with cytokine signaling and production and cytotoxicity at 3 DPI that peaked at 7 DPI (**Figure 4E**; **Supplementary Table 3**).

**Figure 5:**
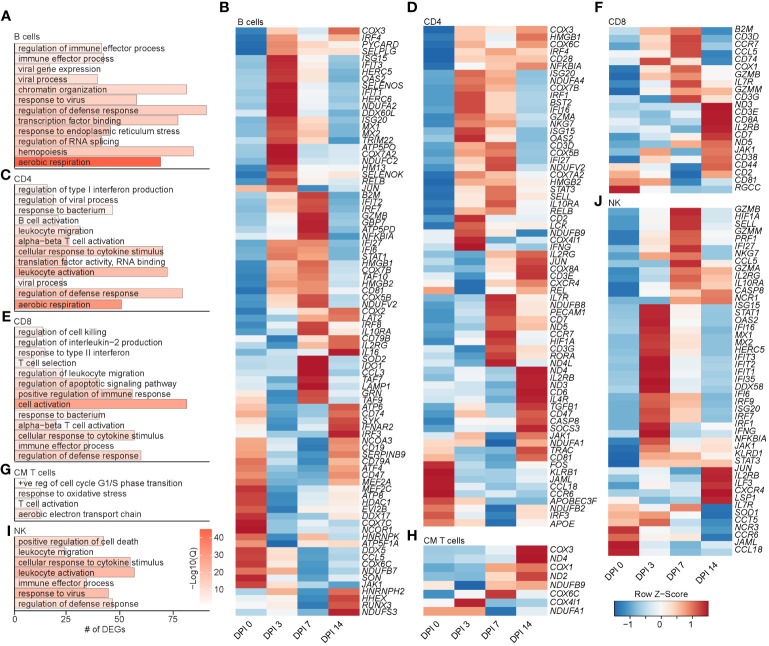
Longitudinal transcriptomic changes within lymphoid BAL cells. Bar plot of GO terms associated with DEGs in B cells **(A)**, CD4 T cells **(C)**, CD8 T cells **(E)**, CM T cells **(G)**, and NK cells **(I)** over time. Length of the bar indicates the number of genes mapping to each GO term, and the intensity of color denotes the −log10(Q-value). Heatmaps of the average expression of the indicated DEGs associated with GO terms in B cell **(B)**, CD4 T cell **(D)**, CD8 T cell **(F)**, CM T cell **(H)**, and NK cell **(J)** clusters. BAL, bronchoalveolar lavage; GO, gene ontology; DEGs, differentially expressed genes.

The frequency of the B-cell and MZ-like/plasmablast clusters increased slightly throughout the acute phase of infection (**Figure 4F**). The expression level of the majority of DEGs in B-cell clusters peaked at 3–7 DPI and mapped to “aerobic respiration” (*ATP5PD*, *COX3*, and *COX7A2*), immune defense processes (*B2M*, *TRIM22*, *CD79B*, *IL16*, *CD19*, *CCL5*, and *HM13*), viral processes (*MX1*, *MX2*, *OAS2*, and *DDX60L*), and processes important for the production of antibodies such as “chromatin organization”, “transcription factor binding”, “response to ER stress”, and “regulation of RNA splicing” (*JUN*, *RELB*, *NFKB1A*, *TAF10*, *SELENOS*, and *SELENOK*) (**Figures 5A, B**). DEGs that peaked at 14 DPI include *CD74* (antigen presentation), *SYK* and *CD19* (B-cell activation), *IRF3* (inflammation), and *ATP6* (cell energy) (**Figure 5B**). Interestingly, some of the downregulated genes are known to play a role in antiviral defense, including *DDX5* and *DDX17*, *CCL5*, and *JAK1* (**Figure 5B**).

The frequency of activated CD4 T cells decreased throughout infection, while that of the GZMA CD4 cluster increased at 3 and 7 DPI (**Figure 4F**). The frequency of EM CD8 T cells increased at 3 DPI, followed by an increase in all CD8 T-cell clusters at 14 DPI (**Figure 4F**). The frequency of proliferating T cells increased at 3 to 7 DPI, with the CM T-cell cluster expanding at 7 DPI in line with prior observations (21). DEGs within CD4 T cells mapped to “aerobic respiration” (*COX3* and *COX7B*) and viral/bacterial processes (“viral process” and “response to bacterium”), as well as T-cell activation (*CD28*, *CD3E*, *CD2*, *CD6*, and *CD7*), migration (*CCR7* and *CCL18*), and “regulation of type I IFN production” (*ISG20*, *IRF4*, and *IFI27*) (**Figures 5C, D**). However, downregulated DEGs within the CD4 T-cell clusters were involved in migration (*CCR6* and *CCL18*) and antiviral responses (*APOBEC3F*) (**Figure 5D**). Upregulated DEGs within CD8 T cells mapped to T-cell effector processes (*CCL5*, *CCR7*, *IL7R*, *GZMM*, and *JAK1*) (**Figures 5E, F**). Finally, DEGs upregulated in CM T cells mapped to oxidative stress (*NDUFB9*), aerobic electron transport chain (*ND2*), and cell cycling (*COX1* and *COX3*) (**Figures 5G, H**).

Prior studies revealed that subjects deficient in NK cells or CD1d molecule experience life-threatening varicella following infection or vaccination (33–35). Thus, we leveraged our scRNAseq dataset to understand the SVV-induced transcriptional changes in NK cell subsets. Longitudinal analysis within NK cell subsets showed that DEGs mapped to cell death (*GZMN*, *GZMB*, and *GZMA*), anti-viral processes (*IFI35*, *IFI16*, *ISG15*, *ISG20*, *MIX1*, and *MIX2*), cellular activation (*STAT1*, *NFKB1A*, *STAT3*, and *JUN*), and migration (*CCL5* and *CXCR4*) (**Figures 5I, J**). Interestingly, the expression of genes important for interferon response and viral processes peaked at 3 DPI, whereas the expression of genes involved in the cytotoxic response (granzymes) peaked at 7 DPI (**Figure 5J**). The expression of genes important for migration (*JAML*, *CCL18*, and *CCR6*) generally decreased (**Figure 5J**). In summary, these data indicate larger changes within the T-cell clusters compared to B cells and NK cells that are accompanied by expansion of CD4 and CD8 T-cell clusters expressing GZMA.

### Distinct transcriptional signature in SVV-infected T cells compared to uninfected T bystander cells

Although the 10x platform allowed us to get an in-depth understanding of the response to acute SVV infection, we were not able to delineate infected versus bystander cells. Therefore, we next used the SMART-Seq2 technology, which allows higher-sequencing depth (23). We FACS-sorted 552 individual BAL T cells and 300 individual BAL macrophages from three different animals at 0 and 7 DPI. We performed qPCR to detect SVV ORF15 transcripts and identify infected and uninfected cells, followed by building full-length cDNA sequencing libraries from each individual sorted cell (**Supplementary Figure 3A**).

**Figure 6:**
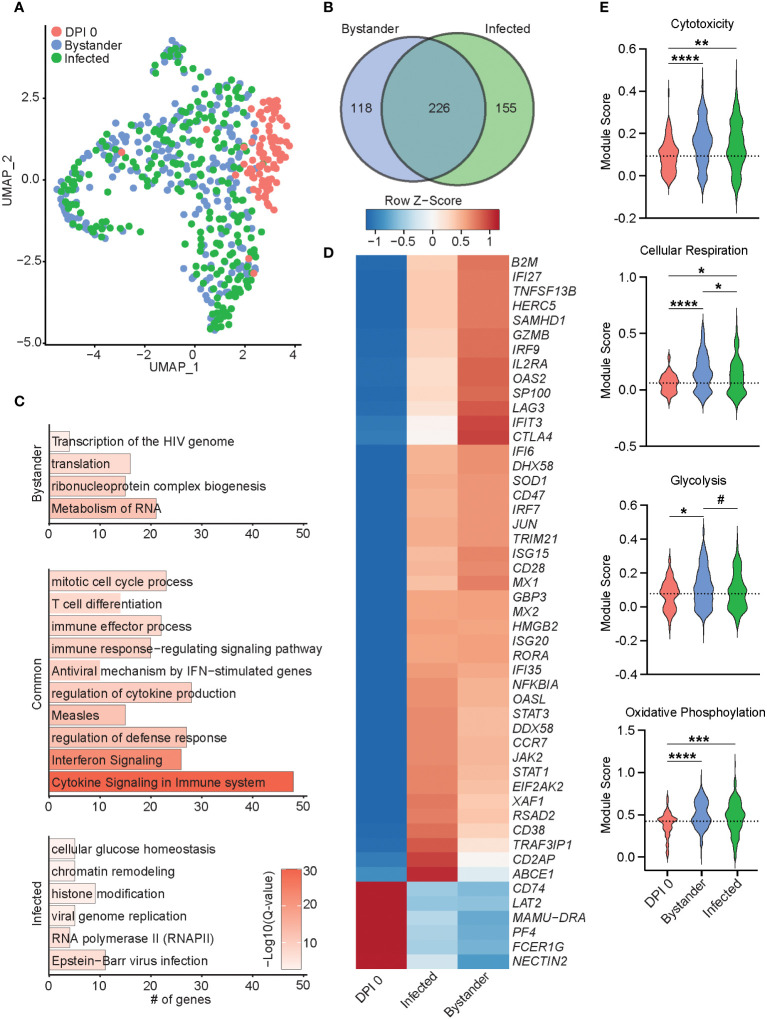
Transcriptional differences of bystander and infected T cells 7 DPI. **(A)** UMAP of 552 T cells colored by timepoint and infection status (0 DPI, DPI 7 bystander, or DPI 7 infected). **(B)** Venn diagram of DEGs between bystander and infected cells relative to 0 DPI. **(C)** Bar plots of GO terms for DEGs unique to bystander cells (top), infected cells (bottom), or common to both (middle). Length of the bar indicates the number of genes mapping to each GO term, and the intensity of color denotes the −log10(Q-value). **(D)** Heatmap of the average expression of select DEGs common to bystander and infected cells. **(E)** Violin plots of the indicated module scores. Dotted line indicates the average value at DPI 0. ^#^p < 0.1, *p < 0.05, **p < 0.01, ***p < 0.001, ****p < 0.0001. DPI, days post-infection; UMAP, uniform manifold approximation and projection; DEGs, differentially expressed genes; GO, gene ontology.

We performed a supervised clustering to compare transcriptomes of infected and bystander cells (**Figure 6A**; **Supplementary Figure 3B**). As expected, SVV ORF transcripts were identified only within the infected cells at 7 DPI (**Supplementary Figure 3C**). The most highly detected ORFs in infected T cells were ORFs 11, 20, and 22 (tegument proteins), ORF 29 (single-stranded DNA binding protein), ORF 33 (viral capsid packaging), and ORFs 61 and 62 (immediate early protein and transcriptional activator), with ORF 61 as the most highly expressed viral gene (**Supplementary Figure 3C–E**).

Next, we performed differential gene expression analysis of 7 DPI infected and uninfected (bystander) T cells compared to 0 DPI, as control cells. The 226 DEGs shared by both infected and bystander T cells mapped to processes associated with cell division and T-cell differentiation (*HMGB2* and CD74), antiviral immunity (*ISG15*, *IFI6*, *GBP3*, *IRF7*, *MX2*, and *RSAD2*), and cytokine response (*LAG3*, *TRAF3IP1*, and *IL2RA*) (**Figures 6B–D**). DEGs downregulated in both bystander and infected T cells included *NECTIN2*, important for herpes simplex virus entry and spread (**Figure 6D**). Gene modules for cytotoxicity, cellular respiration, glycolysis, and oxidative phosphorylation were more highly expressed in infected and bystander cells, while bystander cells had the highest cellular respiration and glycolysis scores (**Figure 6E**). The 118 DEGs unique to the bystander cells mapped to GO terms associated with transcription and translation (“translation”, “ribonucleoprotein complex biogenesis”, and “metabolism of RNA”) (**Figures 6B, C**). The 155 DEGs unique to infected cells primarily mapped to virus infection [“viral genome replication”, “RNA polymerase II”, and “Epstein-Barr virus infection” (*TRIM41*, *OAS1*, *OAS3*, and *PRF1*)], regulation of gene expression [“chromatin remodeling” and “histone modification” (*TUBG1*, *DNMT1*, and *HDAC4*)], and energy metabolism [“cellular glucose homeostasis” (*OXA1L*)] (**Figure 6C**; **Supplementary Figure 3E**). Importantly, genes important for tissue residency (*VIM*, *S100A4*, *MYADM*, and *CXCR6*), as well as those important for organ development, were significantly downregulated in infected cells (**Supplementary Figure 3E**) (36–38). Interestingly, infected T cells were also enriched for expression of TCR variable region 6 (**Supplementary Figure 3E**). Altogether, our data reveal distinct transcriptomic profiles in infected and bystander lung T cells during acute SVV infection with upregulation of genes involved in host defense, chromatin remodeling, histone modification, and energy metabolism in infected cells, potentially reflecting SVV usurping cellular pathways to support viral replication. In addition, the downregulation of tissue homing genes in infected cells could facilitate their trafficking out of the lung.

### Immune response in infected and bystander macrophages during acute SVV infection

**Figure 7:**
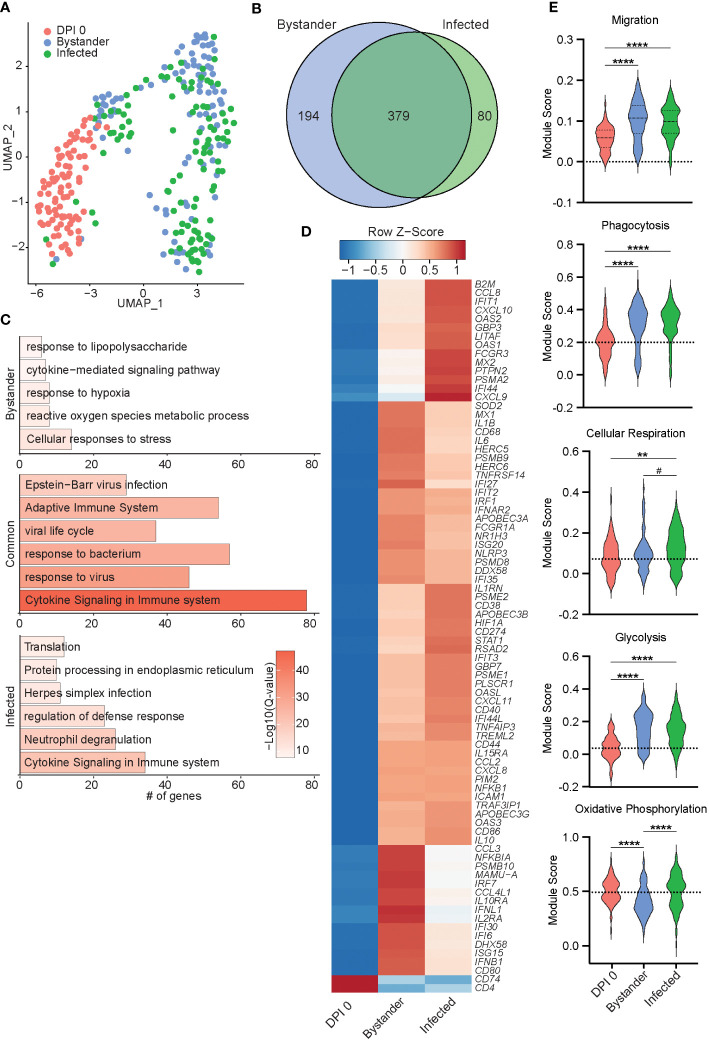
Transcriptional differences of bystander and infected macrophages 7 DPI. **(A)** UMAP of 300 macrophages colored by timepoint and infection status (0 DPI, DPI 7 bystander, or DPI 7 infected). **(B)** Venn diagram of DEGs between DPI 7 bystander and infected cells relative to 0 DPI. **(C)** Bar plots of GO terms for DEGs unique to bystander cells (top) or infected cells (bottom) or common to both (middle). Length of the bar indicates the number of genes mapping to each GO term, and the intensity of color denotes the −log10(Q-value). **(D)** Heatmap of the average expression of select DEGs common to bystander and infected cells. **(E)** Violin plots of the indicated module scores. (Dotted line indicates the average value at DPI 0. ^#^p < 0.07, **p < 0.01, ****p < 0.0001. DPI, days post-infection; UMAP, uniform manifold approximation and projection; DEGs, differentially expressed genes; GO, gene ontology.

SVV transcripts were only detected within infected cells (**Figure 7A**; **Supplementary Figure 4A–C**) with ORF B (DNA cleavage and packaging), ORF 15 (membrane protein), ORF 32 (phosphoprotein), and ORFs 4 and 63 (immediate early proteins) being the most prominent ones (**Supplementary Figure 4B**). Viral transcripts were less abundant in macrophages compared to T cells (**Supplementary Figure 3C**). Differential gene expression analysis between infected and bystander macrophages at 7 DPI relative to 0 DPI identified 379 common DEGs, 194 DEGs were unique to the bystander cells, and 80 DEGs were unique to the infected cells (**Figure 7B**). DEGs common to bystander and infected cells mapped to antimicrobial responses (*HERC5*, *NRLP3*, *APOBEC* genes, *OASL*, and *MX2*), as well as immune processes (*CD86*, *CD274*, *CD44*, *IL1B*, *IL15RA*, *IL10*, and *STAT1*) (**Figures 7C, D**). DEGs unique to the bystander cells mapped to inflammatory processes including “response to lipopolysaccharide”, “response to hypoxia”, and “reactive oxygen species metabolic process” (**Figure 7C**). DEGs unique to infected cells mapped to host defense (*MYD88*, *C3*, *CASP4*, *JAK2*, and *OSM*), viral infection (*RNASE6* and *SVVORFs*), and protein synthesis (*RPL18* and *RPS16*) (**Figure 7C**; **Supplementary Figure 4C**). Interestingly, expression of *FN1*, important for lung tissue residency, was reduced (39), while genes essential for extracellular matrix remodeling were upregulated (*CAPZA1* and *ADAM9*) (**Supplementary Figure 4C**) (40, 41). Additionally, we observed an increase in gene module scores for migration and phagocytosis, as well as cellular respiration and glycolysis, in both the infected and bystander cells compared to 0 DPI cells (**Figure 7E**). Notably, the module score of oxidative phosphorylation was increased in infected cells compared to bystander cells (**Figure 7E**). In summary, these data suggest that SVV infection leads to distinct transcriptional profiles in infected macrophages compared to bystander macrophages.

### Metabolic profile of T cells and macrophages during the SVV infection

The scRNAseq analysis showed that DEGs unique to 7 DPI SVV-infected T cells and macrophages play a role in cellular metabolism (**Figures 6C, E**, **7E**). Studies have shown that activated T cells rely more on aerobic glycolysis and oxidative phosphorylation, while resting T cells primarily use oxidative phosphorylation for energy (42). To investigate this in SVV infection, we performed Seahorse assays to measure cellular glycolysis (ECAR) and mitochondrial oxidative phosphorylation (OCR). Additionally, we assessed the rate of total extracellular acidification via proton production rate (PPR) measurement, as an indicator of overall metabolic activity. Our results revealed a significantly higher ECAR and PPR in T cells and macrophages from SVV-infected animals compared to controls (**Supplementary Figure 5A**). This suggests increased glycolytic activity during infection. Similarly, T cells at 7 DPI activated with PMA/ionomycin had a significantly higher capacity for OCR, ECAR, and PPR compared to 0 DPI, while cells at 14 DPI returned to baseline (**Supplementary Figure 5B**). This pattern aligns with the overall metabolic potential of T cells, which peaked at 7 DPI and normalized at 14 DPI (**Supplementary Figure 5C**). These data suggest that T cells undergo a metabolic shift toward increased glycolysis at 7 DPI during SVV infection, potentially to meet the heightened energy demands associated with activation. Next, we investigated glucose uptake by infected macrophages and T cells compared to controls using 2-NBDG, a glucose analog. We also assessed whether oxidative phosphorylation was affected by measuring mitochondrial activity via MitoTracker staining (43). We observed a significant increase in the percent of 2-NBDG+ T cells, while increased mitochondrial activity was observed only in SVV-infected macrophages (**Supplementary Figure 5D**). Taken together, these data indicate that SVV acute infection leads to a shift toward glycolysis in lung immune cells, which is accompanied by distinct alterations of energy metabolism in T cells and macrophages.

## Discussion

In this study, we used an experimental model where rhesus macaques are infected with SVV that closely recapitulates acute, latent, and reactivated VZV human disease (3, 44, 45) to define the local immune response in the lung and identify pathways that are altered by SVV infection within lung-resident T cells and macrophages. Our study shows that SVV infection results in an infiltration of monocyte-derived macrophages and memory and effector T cells in the lung. Also, we identified transcriptional signatures unique to infected and bystander macrophages and T cells that may explain their ability to act as a Trojan Horse for SVV, notably the decreased expression of tissue-resident markers. Interestingly *NECTIN2* and *ITGAV*, known entry receptors for herpes simplex viruses, were downregulated (46–49). Notably, VZV OKA strain infection and cell to spread were reduced in *ITGAV* knockout cells (50), thus suggesting a potential counter mechanism for T cells to limit varicellovirus entry.

Our single-cell RNAseq data revealed a dynamic shift of BAL innate immune cells during acute SVV infection. Notably, the frequency of the AM subset shrank at 3 DPI, while that of DCs and infiltrating monocyte-derived macrophages (IMs) expanded. Furthermore, the increased frequencies of IMs and DCs in BAL correlate with prior reports of increased levels of chemokines CCL2 and CCL22, known to be important for monocyte and DC recruitment (14, 51, 52). While loss of AM has been reported with some RNA respiratory viruses (53–55), this study is the first one revealing a decrease in AM frequency during acute SVV infection. This shift in macrophage populations can impact the health of the lung, as AM is regulatory M2-like and pro-repair, whereas IM is inflammatory. Consequently, this shift could lead to more tissue destruction after SVV acute infection. Indeed, we previously reported downregulation of genes important for lung development, airspace maintenance, and control of inflammation during acute SVV infection that was accompanied by damage to the alveolar walls and infiltration of inflammatory cells as shown by histology (17). A cluster of IM expressing high levels of ISG was also significantly expanded DPI 3. This aligns with our previous findings of increased IFN-α levels at 3–7 DPI in the BAL (14, 22).

Our study revealed dynamic changes in the lymphoid compartment during SVV infection. The frequency of proliferating T cells and EM CD8 T cells expanded at 3 and 7 DPI, followed by effector CD8 T cells at 14 DPI. This expansion coincided with increased expression of genes linked with T-cell differentiation, activation, signaling, and immune regulation. Notably, the single-cell analysis revealed expansion of both GZMA-expressing CD4 and CD8 T-cell subsets, with CD4 T peaking earlier than the CD8 T-cell subset. Interestingly, GZMA-expressing cells in both subsets harbored signatures of cellular activation and antimicrobial responses, while GZMK-expressing cells leaned toward inflammatory responses. GZMK CD8 T cells are more abundant in tissues affected by inflammatory diseases (56) and expand during the acute phase of COVID-19, particularly in severe cases (57). In contrast, high expression of GZMA by CD4 T cells at baseline was predictive of slower progression of HIV infection and better clinical outcomes (58).

While the frequency of B cells and MZ-like/plasmablasts showed a modest increase following SVV infection, these cells upregulated genes associated with cell activation, immune response, and antibody production. The increased expression of these genes correlated with the production of SVV-specific IgG antibodies in BAL at 14 DPI as previously reported (14). The limited B-cell response suggests that antibodies might play a more crucial role in acute infection rather than controlling established disease, as evidenced by the limited effectiveness of VZV antibody treatment after the development of exanthem (59, 60).

The SMART-Seq technology revealed a higher number of viral genes expressed in T cells. One of the most highly expressed transcripts identified was ORF 61, which plays an essential role in regulating viral gene expression as well as evasion from the type I interferon response (21, 61). Infected macrophages expressed a limited number of viral genes. Among these, viral transactivator ORF 4 and membrane protein ORF 15 were highly expressed, similar to the sensory ganglia during acute infection (20). Differences in viral gene expression profiles between infected T cells and macrophages may be due to the properties of macrophages (virus phagocytosis, less efficient viral replication, and abortive infection), or alternatively T cells may be the preferred target for SVV (62–64). Elucidating the mechanism underlying the differential levels of viral transcripts in these two cell populations will require further investigation.

The SMART-Seq technology also revealed that SVV alters the transcriptional landscape of infected cells. Within infected cells, genes involved in host defense, chromatin remodeling, histone modification, and energy metabolism were upregulated, while genes important for tissue residency were significantly decreased. We propose that the downregulation of tissue homing genes would facilitate the trafficking of infected cells to the sensory ganglia. Interestingly, we also report a potential preference for a specific V beta gene segment in T cells TRBV6–1, which is highly favored by MAIT T cells, which represent up to 10% of airway T cells (65, 66). A recent study revealed that blood MAIT T cells are permissive to VZV infection and retain chemokine receptor expression, suggesting the preservation of their migration capacity (67). We speculate that a similar process could be at play with SVV and lung MAIT T cells. A transcriptional difference with infection status has also been shown with cytomegalovirus, influenza, and herpes simplex virus 1 (68–71). While unique signatures were identified in bystander and infected cells, there was also an overlap in transcriptional profile in both groups relative to 0 DPI. Indeed, the infected and bystander cells are both mounting an immune response, which brings their transcriptional profile to a similar state.

Finally, we found that SVV infection triggered metabolic reprogramming in both T cells and macrophages. Specifically, SVV infection induced glycolysis, potentially due to metabolic shifts to meet the heightened energy demands of the immune cells as reported during influenza infection (72). Interestingly, our transcriptional data revealed that bystander T cells exhibited higher cellular respiration compared to infected T cells and that bystander macrophages have increased cellular respiration and phagocytosis. Therefore, fine-tuning mitochondrial energy resources may provide the host with a double-pronged mechanism to fight SVV infection by increasing energy availability in bystander cells to meet host defense demands and/or reducing energy availability in infected cells to reduce viral replication.

In conclusion, this work expands our understanding of the host response in the lung as well as the re-wiring of T cells and macrophages to act as Trojan Horse for the virus. Given the close homology between VZV and SVV, our findings have important implications for understanding VZV pathogenesis as well as potential complications and secondary infections such as varicella pneumonia or bacterial infections. The study has some limitations. Notably, we were not able to compare the functional potential of infected and bystander cells. Finally, to leverage the single-cell analysis to its fullest, it would be very informative to perform single-cell proteomics to confirm the transcriptional findings presented herein.

## Data availability statement

The datasets presented in this study can be found in online repositories. The names of the repository/repositories and accession number(s) can be found below: PRJNA1091695 (SRA).

## Ethics statement

The animal study was approved by Oregon National Primate Research Center (ONPRC) Institutional Animal Care and Use Committee. The study was conducted in accordance with the local legislation and institutional requirements.

## Author contributions

BD: Data curation, Formal analysis, Investigation, Visualization, Writing – original draft. DM: Formal analysis, Writing – original draft. IM: Conceptualization, Funding acquisition, Supervision, Writing – review & editing.

## Supplementary material

Supplementary Figure 1
**(A)** SVV DNA viral load in BAL supernatant. **(B)** Antigen specific IgG end-point titers in BAL supernatant. **(C)** Percent of proliferating (Ki67+) CD4 and CD8 EM BAL cells. **(D)** Percent of TNFα+IFNγ+ CD4 and CD8 T cells in response to SVV infection in BAL lymphocytes. **(E)** UMAP from colored by DPI. F-H) Heatmap of the average expression of cluster marker genes over time of infection for the indicated GO terms for CCL3 IM **(F)**, SOD2 IM **(G)**, and S100A8 IM **(H)** clusters.

Supplementary Figure 2
**(A-C)** Heatmap of the average expression of cluster marker genes over time of infection for the indicated GO terms for **(A)** GZMA (top), GZMK (bottom) CD4 T cells, **(B)** GZMA (top), GZMK (bottom) CD8 T cells, and **(C)** NK cell clusters.

Supplementary Figure 3
**(A)** qPCR and confirmatory gel of SVV ORF 15 (110bp) expression in BAL cells. SVV ORF 15+ T cells and macrophages were selected for inclusion in SMART-seq2. **(B)** Feature plot of SVVORF expression in T cells. **(C)** Bubble plot of SVVORF expression in 0 DPI, bystander or infected T cells. The size of the bubble denotes the percent of cells expressing the marker, and the color denotes the average expression level of the marker. **(D)** Heatmap of SVVORF expression in 0 DPI or infected T cells and macrophages. **(E)** Bubble plot of DEGs unique to infected T cells. The size of the bubble denotes the percent of cells expressing the marker, and the color denotes the average expression level of the marker.

Supplementary Figure 4
**(A)** Feature plot of SVVORF expression in macrophages. **(B)** Bubble plot of SVVORF expression in 0 DPI, bystander or infected macrophages. The size of the bubble denotes the percent of cells expressing the marker, and the color denotes the average expression level of the marker. **(C)** Bubble plot of DEGs unique to infected macrophages. The size of the bubble denotes the percent of cells expressing the marker, and the color denotes the average expression level of the marker.

Supplementary Figure 5
**(A-C)** Line graph of OCR, ECAR, and PPR in T cells and macrophages from control and SVV+ animals. **(D)** Representative flow gating and bar graphs of the percent of MitoTracker+ and 2-NBDG+ T cells and macrophages.

Supplementary Table 1 Cohort Description.

Supplementary Table 2 Marker genes for clusters and cluster frequencies in **Figures 1**, **2**, and **4**.

Supplementary Table 3 Module score gene lists and average expression tables for **Figures 2**, **4**, **6**, and **7**.
