# Supplementary figures and images for: Transcriptional and functional remodeling of lung-resident T cells and macrophages by Simian varicella virus infection
Source: Front Immunol. 2024 Jun 3;15:1408212. doi: 10.3389/fimmu.2024.1408212 (PMC11180879; doi:10.3389/fimmu.2024.1408212)

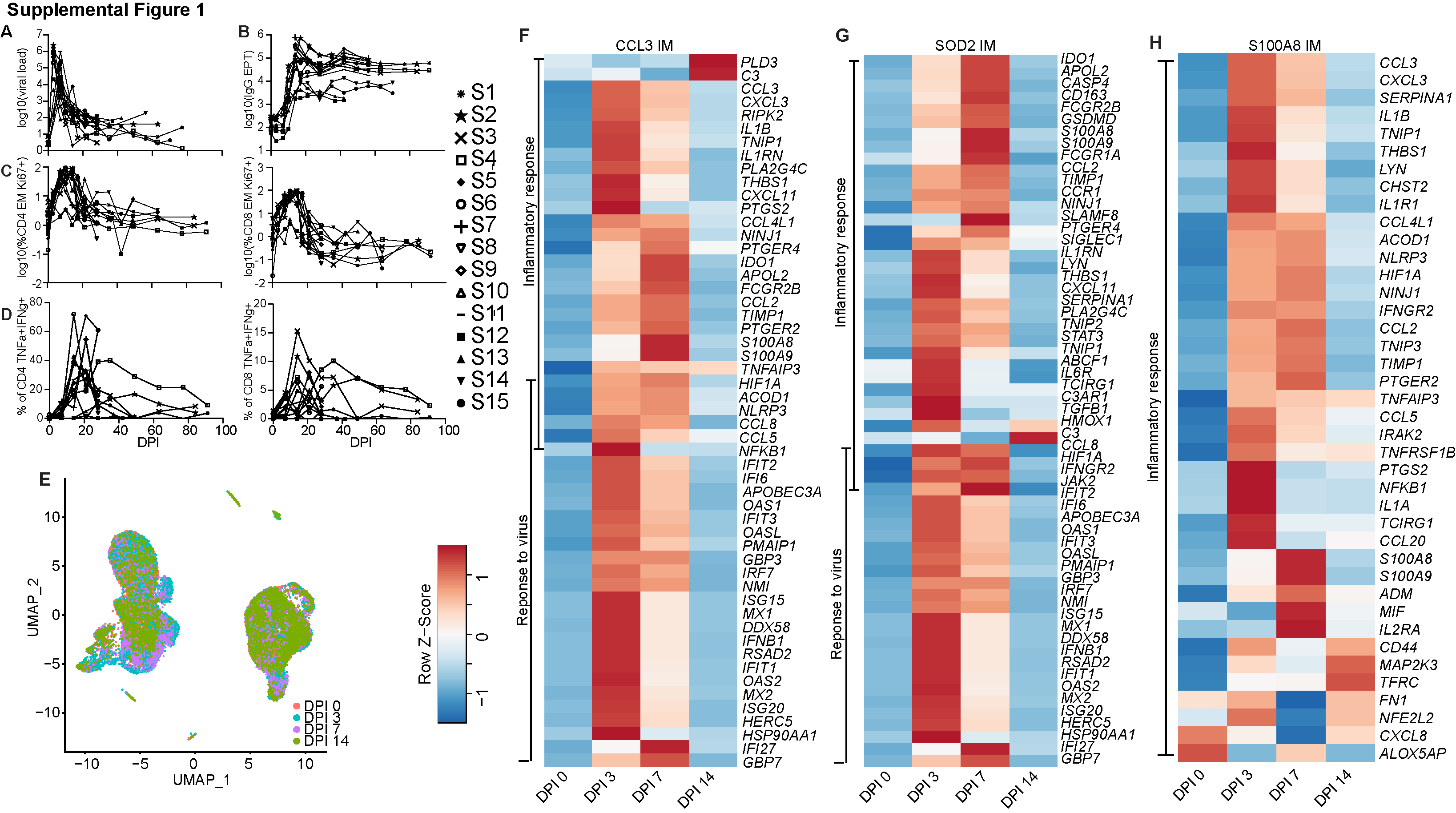

Supplement: Supplementary Figure 1 — (A) SVV DNA viral load in BAL supernatant. (B) Antigen specific IgG end-point titers in BAL supernatant. (C) Percent of proliferating (Ki67+) CD4 and CD8 EM BAL cells. (D) Percent of TNFα+IFNγ+ CD4 and CD8 T cells in response to SVV infection in BAL lymphocytes. (E) UMAP from colored by DPI. F-H) Heatmap of the average expression of cluster marker genes over time of infection for the indicated GO terms for CCL3 IM (F), SOD2 IM (G), and S100A8 IM (H) clusters. [file Image_1.tif]

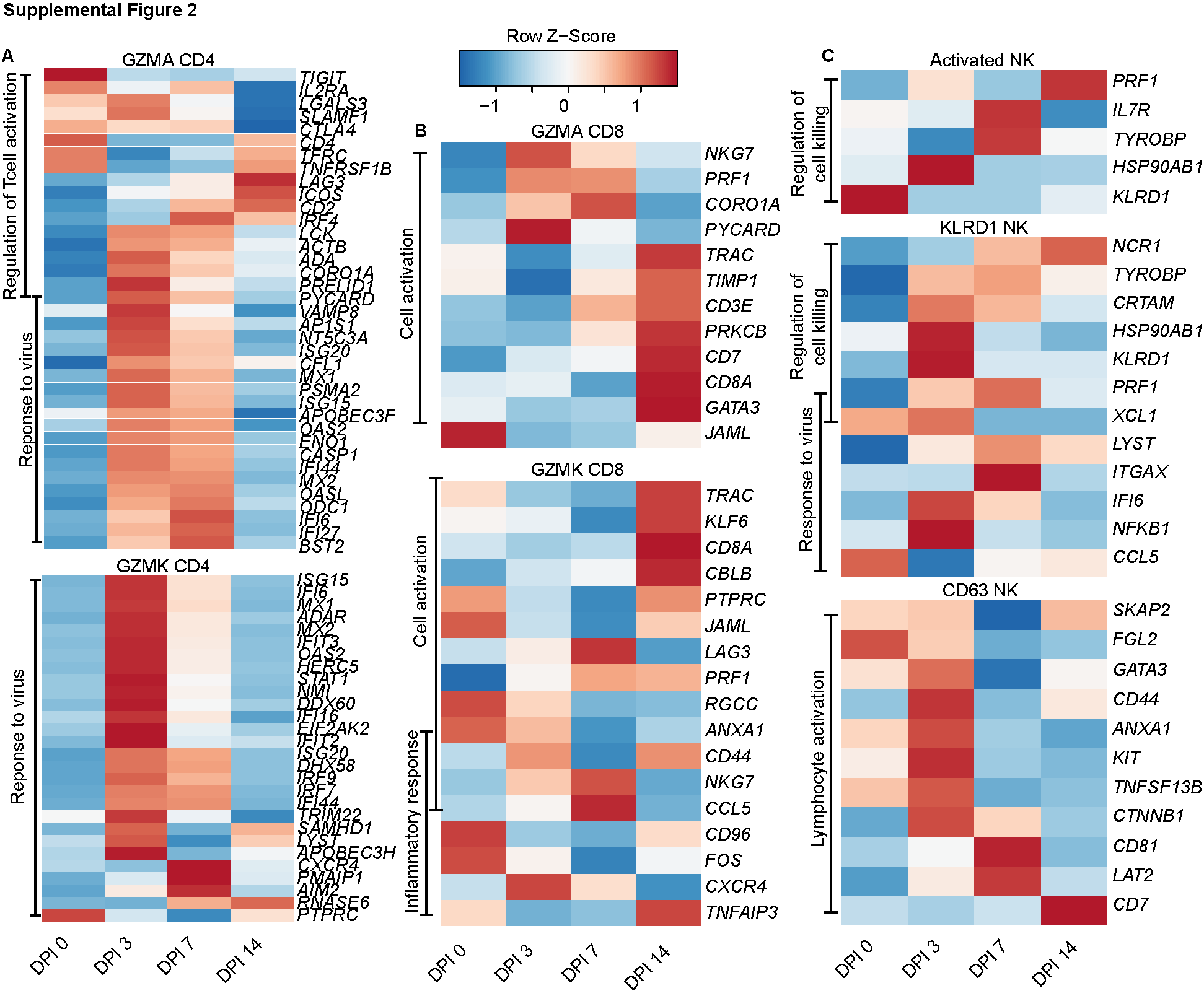

Supplement: Supplementary Figure 2 — (A-C) Heatmap of the average expression of cluster marker genes over time of infection for the indicated GO terms for (A) GZMA (top), GZMK (bottom) CD4 T cells, (B) GZMA (top), GZMK (bottom) CD8 T cells, and (C) NK cell clusters. [file Image_2.tif]

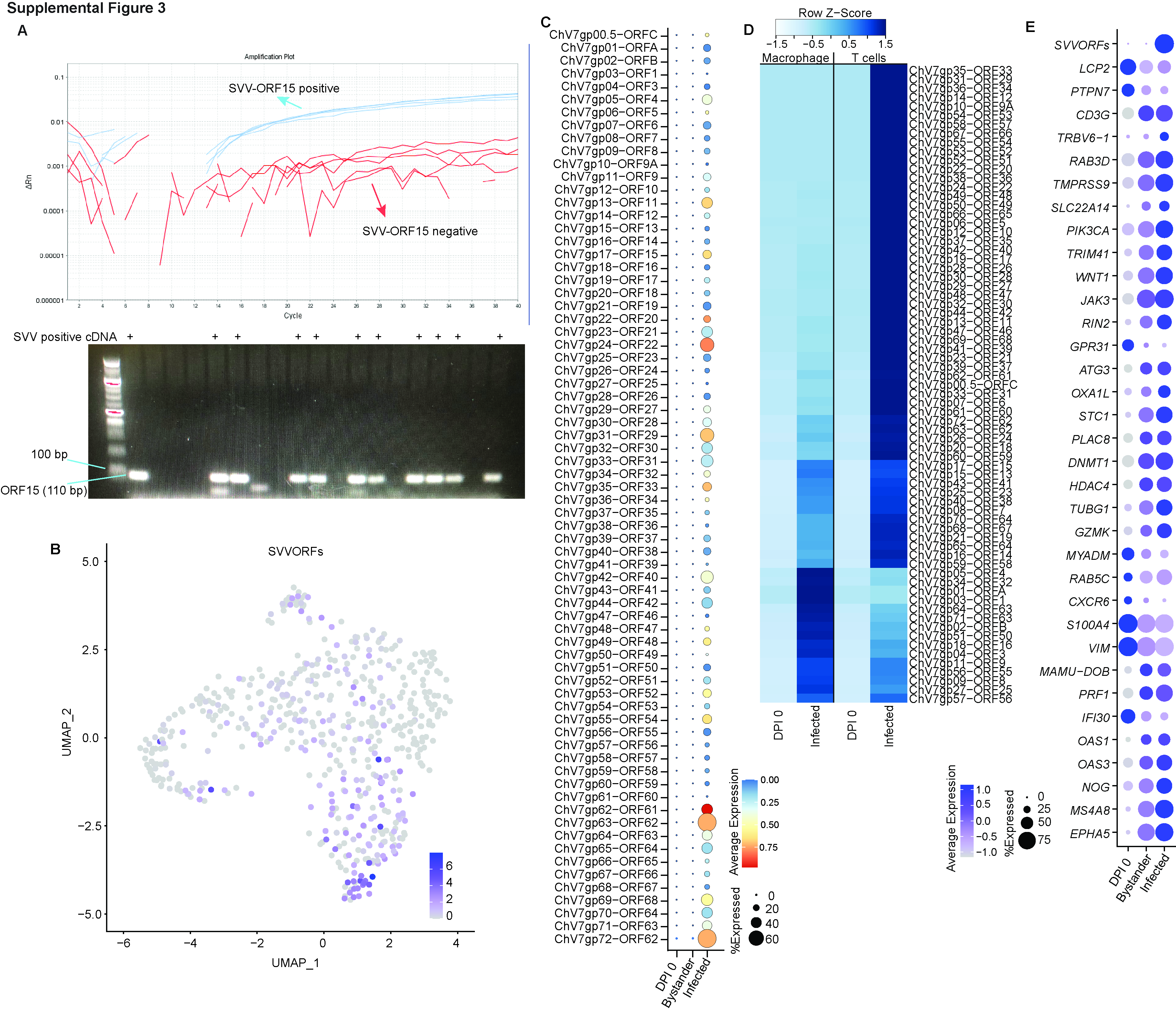

Supplement: Supplementary Figure 3 — (A) qPCR and confirmatory gel of SVV ORF 15 (110bp) expression in BAL cells. SVV ORF 15+ T cells and macrophages were selected for inclusion in SMART-seq2. (B) Feature plot of SVVORF expression in T cells. (C) Bubble plot of SVVORF expression in 0 DPI, bystander or infected T cells. The size of the bubble denotes the percent of cells expressing the marker, and the color denotes the average expression level of the marker. (D) Heatmap of SVVORF expression in 0 DPI or infected T cells and macrophages. (E) Bubble plot of DEGs unique to infected T cells. The size of the bubble denotes the percent of cells expressing the marker, and the color denotes the average expression level of the marker. [file Image_3.tif]

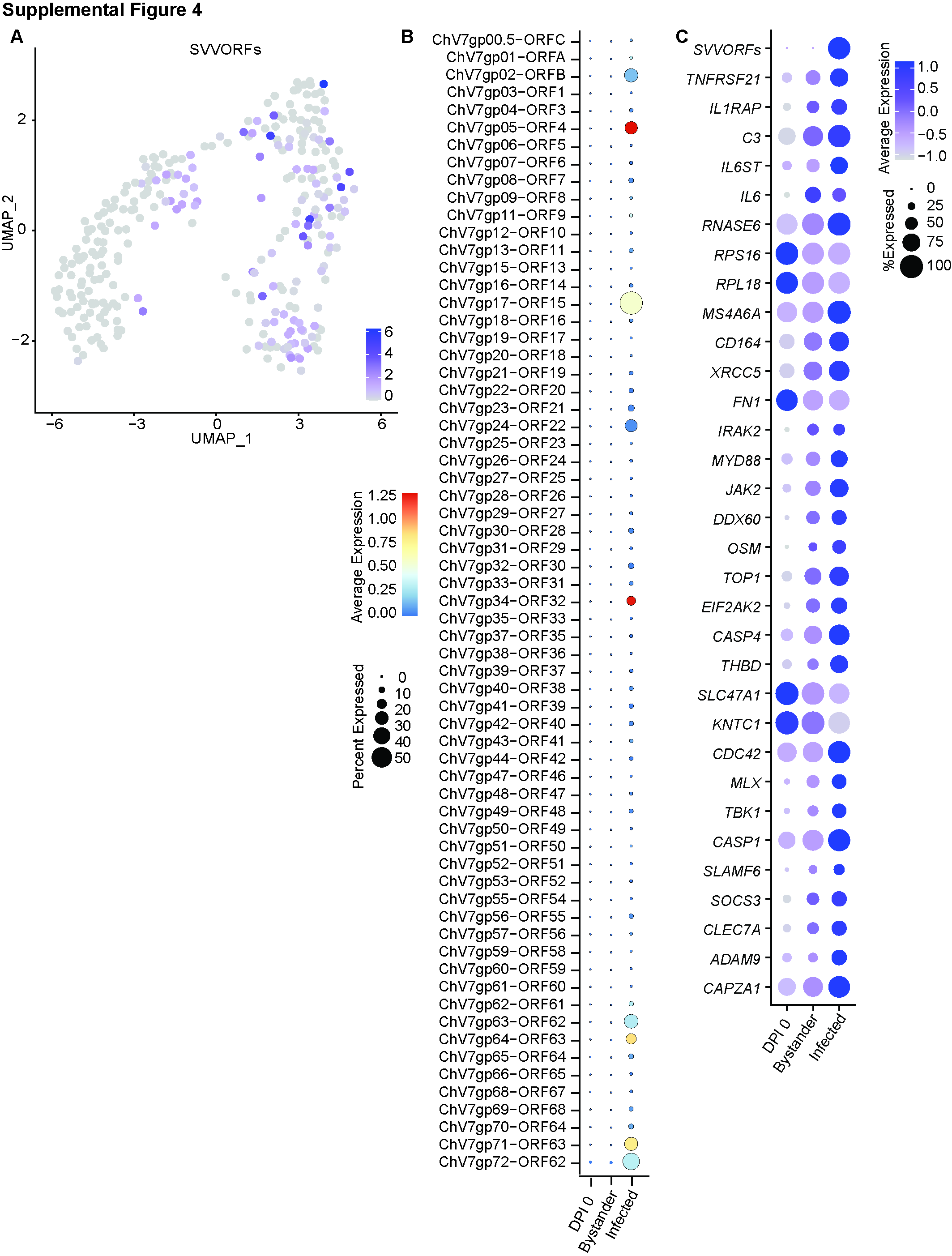

Supplement: Supplementary Figure 4 — (A) Feature plot of SVVORF expression in macrophages. (B) Bubble plot of SVVORF expression in 0 DPI, bystander or infected macrophages. The size of the bubble denotes the percent of cells expressing the marker, and the color denotes the average expression level of the marker. (C) Bubble plot of DEGs unique to infected macrophages. The size of the bubble denotes the percent of cells expressing the marker, and the color denotes the average expression level of the marker. [file Image_4.tif]

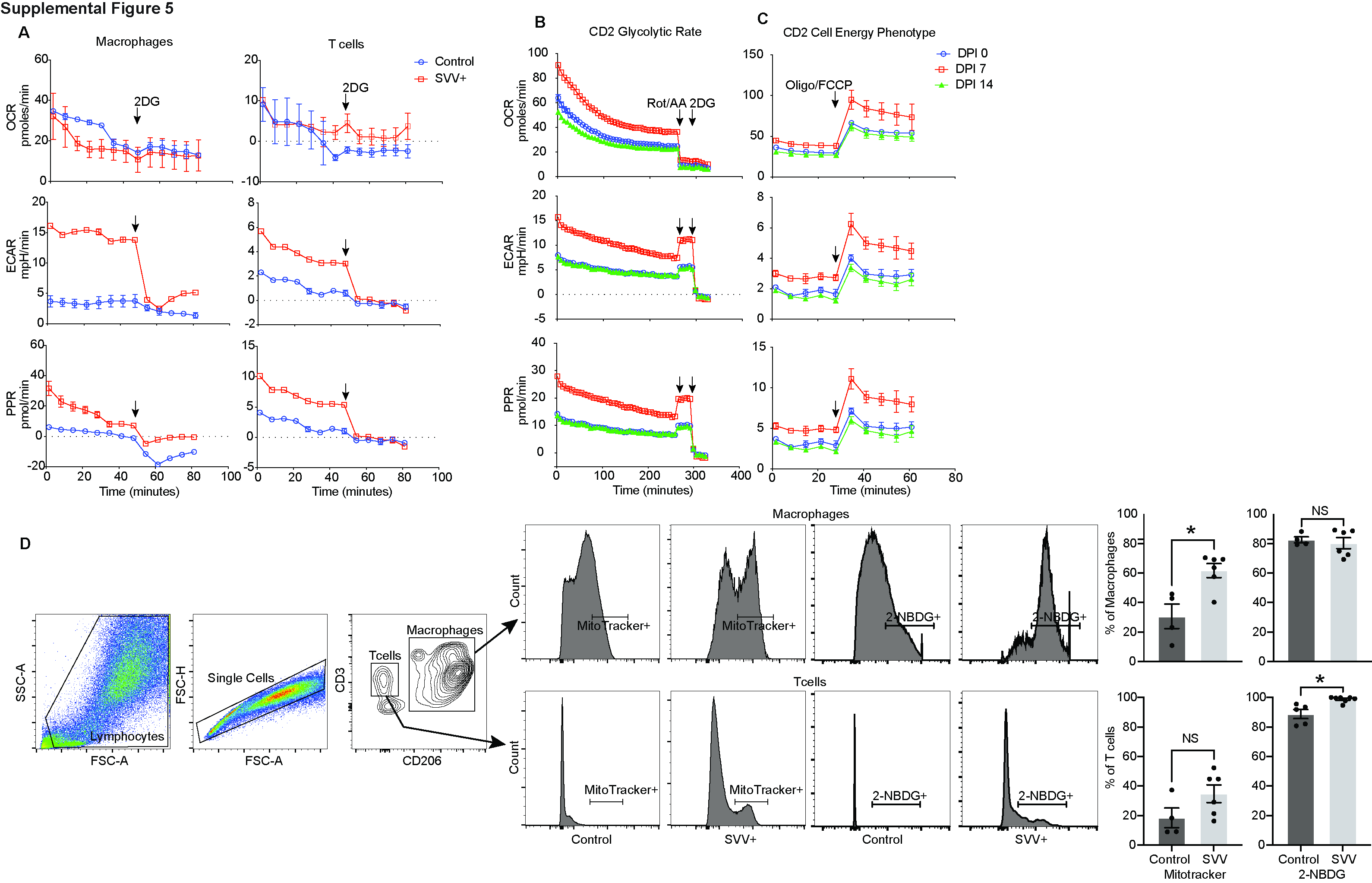

Supplement: Supplementary Figure 5 — (A-C) Line graph of OCR, ECAR, and PPR in T cells and macrophages from control and SVV+ animals. (D) Representative flow gating and bar graphs of the percent of MitoTracker+ and 2-NBDG+ T cells and macrophages. [file Image_5.tif]
